# HPV status represents dominant trait driving delineation of survival-associated gene co-expression networks in head and neck cancer

**DOI:** 10.1038/s41417-022-00577-9

**Published:** 2022-12-27

**Authors:** Ahmed M. Mehdi, Chenhao Zhou, Gavin Turrell, Euan Walpole, Sandro Porceddu, Ian H. Frazer, Janin Chandra

**Affiliations:** 1 Frazer Institute, Faculty of Medicine, The University of Queensland, Woolloongabba, QLD 4102 Australia; 2 Queensland Cyber Infrastructure Foundation Ltd, Facility for Advanced Bioinformatics, Brisbane, QLD 4072 Australia; 3 Princess Alexandra Hospital, Woolloongabba, QLD 4102 Australia; 4 Peter MacCallum Cancer Centre, Radiation Oncology, Melbourne, VIC 3000 Australia

**Keywords:** Oral cancer, Cancer genomics

## Abstract

Integration of high-dimensional tumor gene expression data with clinicopathological data can increase our understanding of disease diversity, enable retrospective patient stratification, and identify new potential biomarkers and therapeutic targets. Using a systems biology approach, we provide a holistic overview of gene co-expression networks in head and neck squamous cell carcinomas (HNSCC). Weighted gene co-expression network analysis of HNSCC RNA sequencing data from 519 patients from The Cancer Genome Atlas (TCGA) was used to determine correlates of 5-year survival, using regression tree-based optimal threshold calculations. Survival-associated gene sets were transformed to gene set scores that were assessed for correlation with clinicopathological data. We identified 8 gene co-expression modules for HNSCC tumors, each of which contained co-expressed genes associated significantly with 5-year survival. Survival-associated co-expression gene signatures correlated dominantly with tumor HPV and p16 status. Network analysis identified that survival was associated with signaling networks of infection, immunity, epithelial-mesenchymal transition (EMT), hypoxia, glycolysis, focal adhesion, extracellular matrix, MYC signaling, autophagy and transcriptional regulation. EMT-associated gene signatures were expressed dominantly in fibroblasts, and cancer-associated fibroblasts were inversely correlated with immune activity. Interestingly, a high Immune Suppression Score based on expression of 21 genes associated with immune inhibition and including immune checkpoints, cytokines and regulatory T cell factors, was also associated with increased survival probability, and was significantly higher in HPV+ HNSCC. Networks associated with HNSCC survival were further associated with survival in cervical cancer, melanoma and lung cancer. This study defines 5129 genes associated with HNSCC survival, organized into co-expressed networks, their correlation with clinicopathological data, and with gene expression data from other malignant diseases, and provides a source for the discovery of biomarkers and novel therapies for HNSCC.

## Introduction

Head and neck squamous cell carcinomas (HNSCC) are the sixth leading cause of cancers, and more than 600,000 new cases are diagnosed yearly worldwide [1].

HNSCC are a diverse group of malignant diseases that are located at various anatomical sites in the mucosal linings of the upper aero digestive tract [2]. Although HNSCC originates from mucosal epithelial cells, it is a heterogeneous disease, originating from different anatomic structures in the oral cavity, oropharynx, nasopharynx, paranasal sinuses, larynx or hypopharynx, and promoted by a range of co-carcinogens, including tobacco, beetle nut, alcohol, and oncogenic human papillomavirus (HPV). Definitive treatment includes either surgery with or without post-operative (chemo) radiotherapy (CRT) or curative CRT and can be associated with significant long-term morbidity. Immunotherapy that targets the immune inhibitory checkpoints PD1/PDL1 has some benefits in recurrent disease with an average response rate of 20–30% [3, 4], but there is still a need for novel therapeutic approaches.

Studies employing high-dimensional sequencing technologies on large patient cohorts, such as The Cancer Genome Atlas (TCGA), have demonstrated genetic mutations contributing to disease initiation and progression, and have been instrumental in identifying new therapeutic targets and biomarkers for patient stratification [5]. While many biomarkers and new therapeutic targets have been suggested based on a targeted mining of sequencing data sets, unbiased identification of disease-associated metabolic processes and pathways should enable a more rational approach to patient stratification and to selection of alternative therapies. We recently described association of a module of immune genes whose expression was associated with increased probability of 5-year survival in cervical cancer, and this immune module included many genes whose expression was associated with immune suppression [6]. In the present study we analyzed the TCGA HNSCC gene expression data, and identified survival-associated co-expressed gene networks, their interrelations and their relationship to clinicopathological data. We further tested the applicability of HNSCC-derived survival-associated gene networks to other types of cancers and identified significant overlap with cervical cancer, lung cancer and melanoma. These data advance our understanding of HNSCC biology, diversity, interrelatedness of signaling pathways, and may guide identification of novel diagnostics, patient stratification, and therapy selection.

## Materials/subjects and methods

### Data collection

Gene expression (HiSeqV2_PANCAN) and clinicopathological data of primary tumors of HNSC (*n* = 519), CESC (*n* = 257), melanoma (*n* = 305), and LUNG cancer (*n* = 917) was obtained from *Xenabrowser (*https://xenabrowser.net/*)*.

The single cell RNA sequencing (scRNA-seq) data of HNSCC tumor samples was derived from Gene Expression Omnibus (GEO) DataSets (GSE103322) [7].

### Weighted gene co-expression network analysis

Weighted gene co-expression network analysis (WGCNA) was performed as previously described [6], using the *WGCNA* software package in R [8]. Briefly, the similarity between each pair of genes across all samples in TCGA-HNSCC was determined using a Pearson correlation method. We then transformed the correlation values to an adjacency matrix by raising the similarity power to 6. By using the adjacency matrix, we computed a topological overlap matrix (TOM) to determine the topological similarity and a corresponding dissimilarity matrix (1-TOM) to form clusters. The hclust function was used to perform hierarchical clustering using the dissimilarity matrix. The outliers were determined using the *flshclust* software package in R [9]. Highly distinct modules were uncovered by using the dynamic tree-cutting algorithm. The colors were randomly assigned to each module. By using the expression values of all genes in each module, eigen gene expression per module (ME) was calculated representing the first principal component for each module. The clinical features were correlated with ME of each module.

### Kaplan–Meier survival analysis

Kaplan–Meier survival analysis (KMSA) was based on a regression tree-structured statistical model [10] and was performed for each gene of each WGCNA module. Specifically, for each gene, an optimal threshold was identified using recursive binary partitioning to establish the association between gene expression and time to death.

Therefore, the method stratifies the patients into two survival groups termed as “high- expression” and “low-expression”. The KMSA was performed using the R software packages *Survminer* and *Survival* with the default settings of Rho = 0 to extract a log rank test *p*-value for overall survival up to 1825 days (5 years), with *p*-values <0.05 considered significant.

### Pathway and interaction analysis

Significant survival-associated genes of each WGCNA module were sorted into two lists; one where high expression of genes was associated with increased 5-year survival (IS), and the other where high expression of genes was associated with decreased 5-year survival (DS) (Supplementary File 1).

Each gene list was subjected to pathway analysis using Enrichr (https://maayanlab.cloud/Enrichr/) and protein-protein-interaction analysis using the Search Tool for the Retrieval of Interacting Genes (STRING) (https://string-db.org/), using the highest confidence threshold of 0.9. The resulting interaction network was imported into Cytoscape for better visualization.

### Analysis of scRNA-seq data of HNSCC tumor samples

The raw count matrix and annotation of cancer and non-cancer cell clusters were downloaded, processed, and visualized using the *Seurat* R package (v3.0.5) [11]. The fibroblast cluster was further divided into three subpopulations (CAF, myofibroblasts, and resting fibroblast) based on the expression of specific genes as described in the original publication [7].

### Gene set enrichment analysis

Gene set enrichment analysis was performed using the *AUCell* pR ackage [12]. *AUCell* calculates the area under the curve score for each cell to determine whether the input gene signature is enriched within the top-ranking genes (ordered by their expression values) for each cell. A total of 55 myogenesis genes and a total of 44 EMT genes obtained from pathway analysis were used as input gene sets. For each cell in the HNSCC scRNA-seq dataset, enrichment scores of both gene sets were calculated independently. Enrichment scores of cells within the same cell type were then compared with those of cells from different cell types. A threshold defining which cell was highly enriched for the input gene sets was determined by the *mixtools* R package [13].

### Score development

WGCNA-derived survival-associated gene lists in each module (Supplementary File 1) were translated into scores using a method recently established by Foroutan and colleagues [14].

Briefly, each TCGA-HNSCC sample was ranked by increasing abundance for each gene using the ‘rankgenes’ function in the *Singscore* software package [14], and a mean rank was calculated for each sample in TCGA-HNSCC data. The mean rank was further normalized against ranks of all samples in the data. Lower and upper quartiles of scores were subsequently compared for 5-year survival using KMSA.

### Immune-suppression analysis

A knowledge-based immune suppression gene list containing 50 genes, including immune inhibition checkpoint genes, regulatory T cell genes and immune-suppressive cytokines was tested for association with survival using KMSA. Genes where high expression was associated with increased survival were summarized into an “Immune Suppression Score” (Supplementary File 1).

### Trait relationships

Relationships between clinicopathological data and modules eigengene expression; and between module scores were established using Pearson R correlation analysis (PRCA).

### Computer availability

Custom computer code can be provided upon request to the corresponding author.

## Results

### Multiple gene co-expression networks are associated with survival in HNSCC

**Fig. 1:**
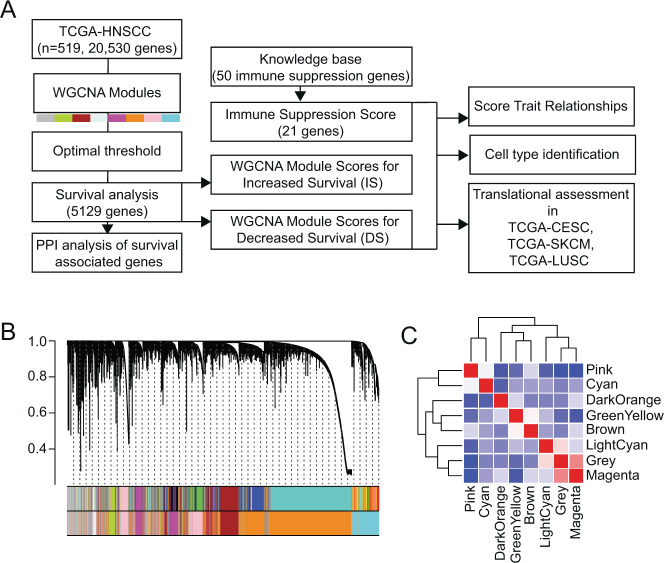
Gene network delineation in HNSCC. **A** Flow chart describing analysis pipeline. **B** Weighted gene co-expression network analysis (WGCNA) was used to delineate gene networks in the TCGA HNSCC data. Clustering of module eigengenes with a threshold of 0.6 resulted in 8 modules. **C** Correlation analysis between WGCNA modules.

**Fig. 2:**
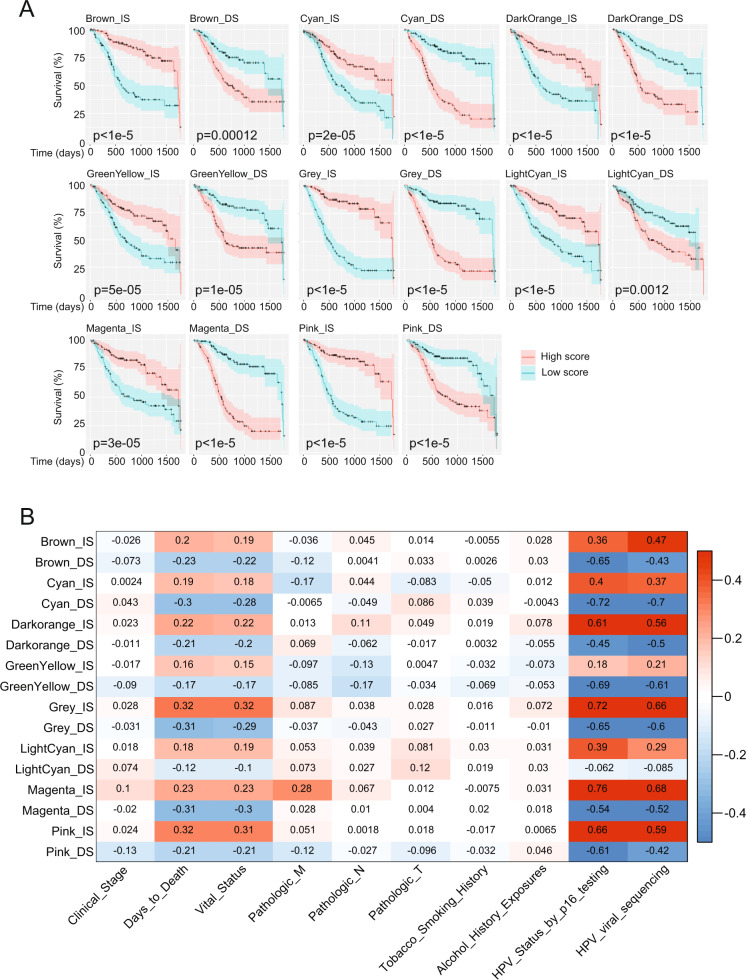
Survival-associated gene networks and their relationship with clinical data in HNSCC. **A** Genes in WGCNA modules were analyzed for association with survival. Significant genes were divided into gene lists where high expression was associated with increased survival (IS) or decreased survival (DS), and were subsequently summarized as _IS and _DS scores. Scores were validated for association with survival by comparing upper and lower quartiles. **B** WGCNA module-derived scores were correlated with clinicopathological traits.

We performed weighted gene co-expression network analysis (WGCNA) across 20,530 genes for 519 samples comprising the TCGA HNSCC primary tumor cohort to determine central signaling networks associated with HNSCC prognosis (Fig. 1A) We identified 8 co-expression modules (Fig. 1B) with only weak inter-module correlation (Fig. 1C). Expression of individual genes from each module was assessed as a predictor of 5-year survival, using recursive binary partitioning, allowing identification of the most discriminatory level of gene expression for each gene (Fig. 1A). Overall, across the 8 modules, we identified 2305 genes where high expression was associated with increased survival (IS), and 2824 genes where high expression was associated with decreased survival (DS), resulting in 16 gene lists (Supplementary File 1). We summarized the 16 gene lists as module scores [14], and verified that the top and bottom quartiles of scores were predictive of survival (Fig. 2A). We observed that multiple WGCNA module-derived survival-associated gene lists were significantly enriched for central pathways and protein–protein interaction (PPI) networks (Table 1).

**Table 1:** WGCNA identifies multiple central networks associated with survival in HNSCC.

Module	Expression associated with increased/decreased survival	Number of genes	Pathway enrichment	PPI enrichment *p*-value
Brown	Increased	191	n.s.	n.s.
	Decreased	173	Protein secretion; Apical junction;	0.00201
Cyan	Increased	638	Cytokine-cytokine receptor interaction; T cell receptor signaling; Natural killer cell mediated cytotoxicity;	<1.0e−16
	Decreased	309	Lysosome	0.00839
Dark orange	Increased	570	Herpes simplex virus 1 infection; Generic Transcription Pathway *Homo sapiens* R-HSA-212436;	4.27e−05
	Decreased	1565	Myc Targets V1, Myc Targets V2, mTORC1 signaling; Metabolism of proteins *Homo sapiens* R-HSA-392499;	<1.0e−16
Green Yellow	Increased	148	Estrogen response early and late; Oxidation by Cytochrome P450 WP43	<1.0e−16
	Decreased	42	O-linked glycosylation Homo sapiens R-HSA-5173105; Gap junction;	0.000144
Grey	Increased	261	Herpes simplex virus 1 infection; Generic Transcription Pathway Homo sapiens R-HSA-212436;	n.s.
	Decreased	246	Complement and coagulation cascades; Apical complex;	9.8e−13
Light Cyan	Increased	36	n.s.	n.s.
	Decreased	3	n.s.	n.s.
Magenta	Increased	337	E2F targets; G1 to S cell cycle control WP45; Retinoblastoma gene in cancer WP2446;	2.22e−16
	Decreased	25	E2F targets; G2-M Checkpoint	0.0277
Pink	Increased	124	n.s.	n.s.
	Decreased	461	Myogenesis; Epithelial-Mesenchymal Transition; Hypoxia;	<1.0e−16

PPI was analyzed using STRING-DB with the highest confidence of 0.9; n.s. not significant with adjusted *p*-value above 0.05.

We performed correlation analysis between the WGCNA module-derived scores and clinical traits (Fig. 2B) and determined that the scores were not significantly associated with clinical stage, pathological tumor/node/metastasis (T/N/M) stage, smoking or alcohol consumption. However, there were positive correlations for IS genes, and negative correlations for DS genes, with days to death and vital status (0 = dead, 1 = alive). Expression of p16 has been used to determine HPV status indirectly [15], and a recent study identified HPV viral gene reads in the TCGA sequencing data [16], providing direct evidence of HPV status. The strongest correlations with the WGCNA module-derived scores were with p16 status and with detection of HPV viral sequences. Scores of IS genes were positively correlated with p16 and HPV status, and scores of DS genes were negatively correlated with HPV status, in keeping with the observation that HPV+ HNSCC have a better average prognosis than HPV− HNSCC [17, 18]. Surprisingly, recorded tobacco use did not correlate with any of the WGCNA module-derived survival-associated scores. We detected the highest correlations of HPV status with the Magenta_IS score. This module includes 337 genes with high PPI significance, and is enriched in zinc finger transcription factors associated with ‘Herpes simplex virus 1 infection’ as well as the ‘E2F target pathway’ and ‘Cell cycle control’. Interestingly, high expression of E2F and its target genes is commonly associated with poor epithelial cancer prognosis [19]. HPV E7 oncoprotein binds to the retinoblastoma protein (Rb), thereby releasing E2F proteins to initiate cell cycle [20]. However, some E2Fs act as tumor suppressors, including E2F8, a gene included in the Magenta_IS score. E2F8 also plays an important role in the DNA damage response by preventing DNA synthesis when there is DNA damage, possibly resulting in less aggressive tumor growth and hence a better prognosis.

A second module score that was strongly correlated with HPV status was the Grey_IS score. This score was built on 261 IS genes, which were also enriched in zinc fingers associated with ‘Herpes simplex virus 1 infection’ and ‘Generic Transcription Pathways’. Further, we detected many zinc fingers (176 ZNFs, 11 ZFP, others) across multiple WGCNA modules (DarkOrange, Grey, Magenta, Pink). Given their role in regulating gene expression, it is not surprising that zinc finger proteins act as tumor promoters or suppressors [21]. Zinc finger proteins hence represent a largely untapped source for biomarker and target discovery.

**Fig. 3:**
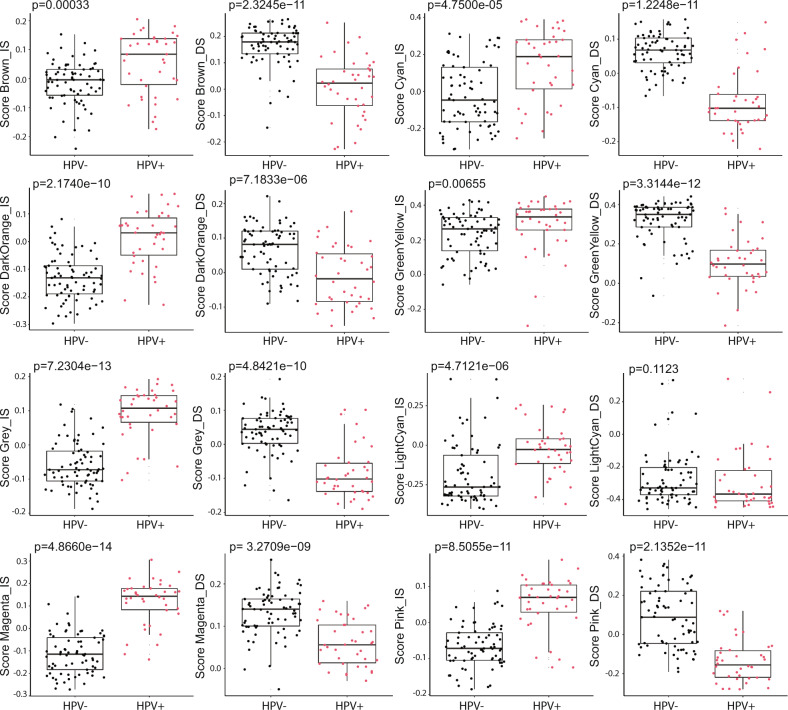
HPV status is dominant trait driving delineation of survival-associated gene co- expression networks in head and neck cancer. WGCNA module scores were compared by HPV_p16 status (negative: *n* = 73, positive: *n* = 38). Boxplots indicate median and interquartile range. Homogeneity of variance was tested using Levene test. *P*-values are based on either student *t*-test when score values were normally distributed, or Wilcoxson rank-sum test when score values were non-normal distributed.

WGCNA module-derived scores were generally significantly different between HPV− and HPV+ HNSCC (Fig. 3), suggesting that HPV status is the main determinant of survival-associated co-expression gene signatures, and reinforcing the observation that HPV+ and HPV− HNSCC are different disease entities [22].

In the remainder of this study, we focused on three of the WGCNA module-derived gene networks, Cyan_IS, Pink_DS and DarkOrange_DS.

### An immune network is associated with increased survival probability in HNSCC

**Fig. 4:**
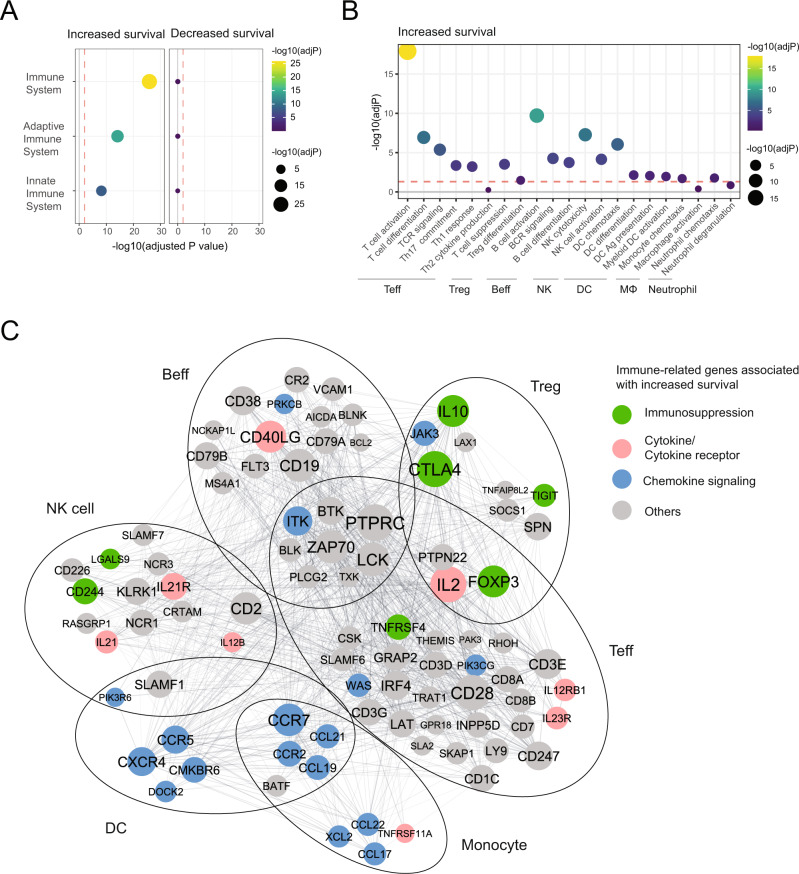
An immune network is associated with increased survival probability in HNSCC. **A** 638 WGCNA Cyan module genes associated with increased survival and 309 genes associated with decreased survival were analyzed separately in Enrichr using the Reactome Pathway Database. Dot plot shows enrichment patterns of three representative immune-related pathways. The red dashed line represents a cut-off for the adjusted *p*-value (adj*P* = 0.01). **B** 638 WGCNA Cyan module genes associated with increased survival were further analyzed in Enrichr using the GO Biological Process Pathway Database. Dot plot shows enrichment patterns of different cell-type-associated immune pathways (effector T cell: Teff; Regulatory T cell: Tregs; effector B cell: Beff; NK cells; DC; Macrophage: MΦ; Neutrophil). The red dashed line represents a cut-off for the adjusted *p*-value (adj*P* = 0.05). **C** A total of 92 WGCNA Ccyan module genes associated with increased survival identified in the different cell-type-associated immune pathways from (**B**) were further analyzed using STRING (https://string-db.org/) and the resulting interaction network was imported into Cytoscape for better visualization. Genes derived from the same cell-type-associated immune pathways were grouped together. Node size indicates the degree of connectivity determined by “active interaction sources” as described in STRING. Non-connected genes were removed from the network. Nodes with different colors represent genes encoding immunosuppressive molecules (green), cytokine/cytokine receptor (pink) and chemokines and their signaling molecules (blue).

The WGCNA Cyan module consisted of 638 IS genes and 309 DS genes (Table 1, Supplementary File 1). Cyan_IS genes were significantly enriched for the Reactome pathways ‘Immune System’, ‘Adoptive Immune System’, and ‘Innate Immune System’, whereas no pathway enrichment was observed for Cyan_DS genes (Fig. 4A). The IS genes were further enriched for cell-type-associated immune pathways, including regulatory and effector T cells, B cells, NK cells, DCs, macrophages and neutrophils (Fig. 4B). PPI analysis determined genes of highest connectivity, including *CTLA4*, *FOXP3*, *PTPRC*, *LCK*, *IL2*, *CD19*, and *CD40LG* (Fig. 4C). The network further contained multiple genes indicative of immune suppression such as *TIGIT*, *LGALS9*, *TNFRSF4,* and *CD244*. Together, these data demonstrate that a rich immune network is associated with increased survival probability in HNSCC, and that this immune network includes genes indicative of immune suppression.

**Fig. 5:**
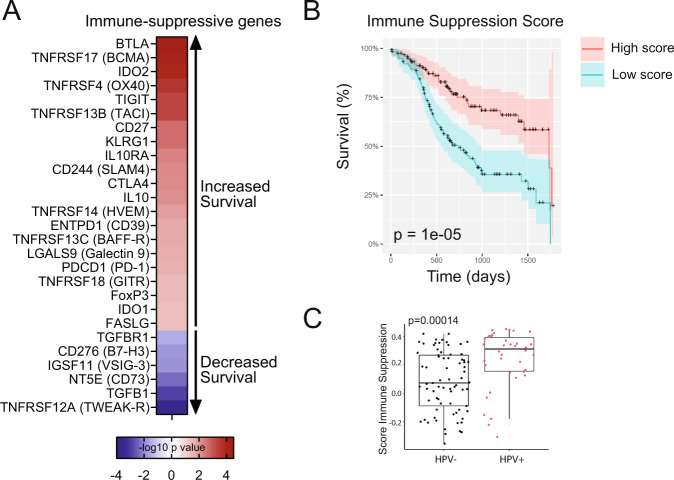
High expression of immune suppression genes is associated with increased survival probability in HNSCC. **A** A knowledge-based list of 50 immune suppressive genes was analyzed for association with 5-year survival probability. *p*-values were −log10 transformed and genes for which high expression was associated with decreased survival were transformed to a negative value. **B** An Immune Suppression Score was built based on 21 immune suppression genes shown in (**A**) where high expression was associated with increased survival. Each subject was assigned an Immune Suppression Score and 5-year survival was analyzed by comparing upper and lower quartiles of the score. **C** The Immune Suppression Score was compared by HPV_p16 status (negative: *n* = 73, positive: *n* = 38). Boxplots indicate median and interquartile range.

We previously demonstrated that high expression of 20 immune suppressive genes was associated with increased survival probability in cervical cancer [6]. To determine which immune suppression genes are associated with survival in HNSCC, we performed survival analysis using a knowledge-based list of 50 immune suppression genes, and identified 21 IS genes and 6 DS genes (Fig. 5A). The majority of these genes overlapped with those observed in the cervical cancer study [6]. Furthermore, *TNFRSF12A* (TWEAK-R), *NT5E* (CD73), and *TGFB1/TGFBR1* were genes where low expression was significantly associated with increased survival in both cervical cancer and HNSCC, demonstrating that high expression of these immune genes is detrimental to outcome. We built an Immune Suppression Score based on the 21 IS immune suppression genes, and demonstrate that a high Immune Suppression Score is associated with increased 5-year survival probability (Fig. 5B), similar as to our previous observations in cervical cancer [6]. Furthermore, the Immune Suppression Score was significantly different between HPV+ and HPV− HNSCC, where HPV+ HNSCC generally displayed higher Immune Suppression Scores (Fig. 5C).

### An EMT network is associated with decreased survival probability in HNSCC

**Fig. 6:**
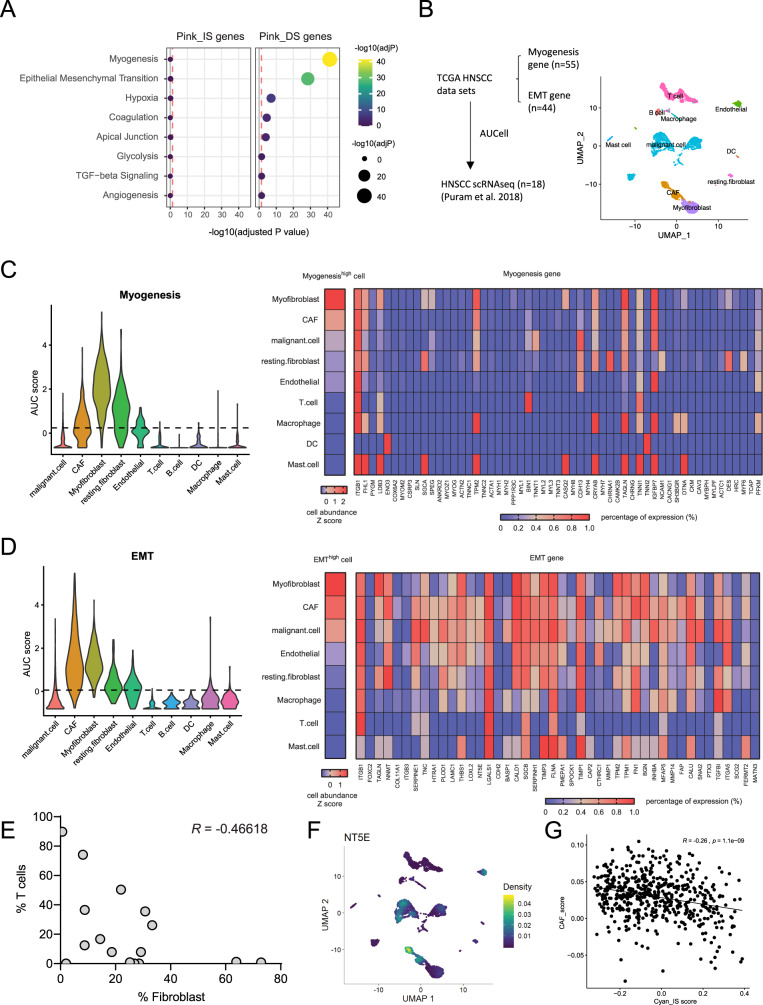
An EMT network is associated with decreased survival probability in HNSCC. **A** Genes of the Pink_IS score (*n* = 124) and the Pink_DS score (*n* = 461) were analyzed for pathway enrichment. **B** Enrichment scores of ‘Myogenesis’ and ‘Epithelial Mesenchymal Transition’ (EMT) were calculated for each cell of a published HNSCC scRNAseq dataset [7] using the *AUCell* package. **C, D** Myogenesis (**C**) and EMT (**D**) gene set enrichment scores for each cell type were visualized as Violin plot (left). Heatmaps (right) show ranking of Myogenesis (**C**) and EMT (**D**) enriched cell types (gene enrichment scores above the threshold as determined by *Mixtools* package) and expression of pathway specific genes across cell types. **E** Pearson correlation analysis was performed between the proportion of T cells and the proportion of fibroblasts of the HNSCC scRNAseq data [7]. **F** Density plot depicts expression of *NT5E* (CD73) across cell types [7]. **G** Correlation between CAF_score and Cyan_IS score in TCGA-HNSCC.

The WGCNA Pink module consisted of 124 genes associated with IS and 461 genes associated with DS (Table 1, Supplementary File 1). In this module, IS genes were not significantly enriched for particular pathways. In contrast, DS genes were enriched in the’Myogenesis’,’Epithelial-mesenchymal transition’ (EMT), and ‘Hypoxia’ pathways (Table 1, Fig. 6A). To determine which cell types expressed DS genes, we used single-cell RNA sequence data (scRNAseq) transcriptomic reads of ~6000 cells from 18 primary HNSCC biopsies [7] (Fig. 6B). We performed gene set enrichment analysis using the DS-associated genes of the myogenesis and the EMT pathways, and found that myofibroblasts, cancer-associated fibroblasts (CAFs), resting fibroblasts and endothelial cells showed the highest enrichment for genes associated with myogenesis and EMT (Fig. 6C, D). While noting that a scRNAseq dataset derived from 18 tumor biopsies is not powered to confidently define relationships, we observed a moderate negative correlation between numbers of T cells and fibroblasts (Fig. 6E), and that CAFs expressed high levels of *NT5E* mRNA (Fig. 6F). *NT5E* encodes the ecto-5′-nucleotidase CD73 which converts adenosine monophosphate (AMP) to adenosine and is thereby known to contribute to immune suppression by inducing regulatory and anergic T cell responses [23, 24]. CAFs are a major site of CD73 expression and mediate T cell suppression in colorectal cancer [25]. We further developed a CAF score based on a CAF gene expression signature [26] and found that a high CAF score was significantly associated with decreased survival probability in the TGCA HNSCC data (*p* = 0.00911, data not shown), and was inversely correlated with the immune module derived Cyan_IS score (Fig. 6G).

### A MYC network is associated with decreased survival probability in HNSCC

The WGCNA DarkOrange module consisted of 570 IS genes and 1565 DS genes (Table 1, Supplementary File 1). A significant number of DS genes were enriched in the ‘MYC targets V1 and V2 pathways’, ‘mTORC1 signaling’, ‘Unfolded protein response’, ‘Oxidative phosphorylation’, ‘Glycolysis’ and ‘Hypoxia’ pathways. MYC is an oncogenic transcription factor that controls cell proliferation, apoptosis, telomerase activity and VEGF-mediated angiogenesis [27, 28]. MYC targets scores have also been associated with cancer aggressiveness in breast cancer [29]. This module also contained a large number of ribosomal genes, indicating increased biosynthesis, as well as a significant number of proteasome subunit genes, potentially suggesting increased levels of autophagy, which was further supported by the presence of the essential autophagy genes *ATG5*, *ATG12*, *BECN1* and vacuolar protein-sorting-associated (VPSA) proteins in this module. The 1565 co-expressed genes of this module may thus provide a resource for therapeutic target identification.

### Key HNSCC-derived survival-associated co-expression networks apply to other cancer types

**Fig. 7:**
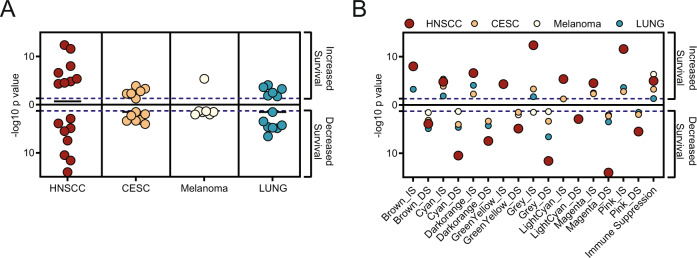
Parallels between HNSCC survival-associated gene networks and CESC, Melanoma, and Lung cancer. The WGCNA module-derived Scores and the Immune Suppression Score were tested for survival association in the TCGA datasets for cervical cancer (CESC), melanoma (SKCM) and lung cancer (LUNG). Data shows −log10 transformed *p*-values of KMSA when top and bottom quartiles of scores were compared. Only significant KMSA *p* values were transformed and plotted. **A** Summary where each data point represents a −log10 transformed *p*-value of one individual WGCNA module-derived score. **B** Each KMSA −log10 transformed *p*-value is shown for each individual score. Dotted line represents statistical significance threshold of −log10 transformation of *p* = 0.05.

We have previously shown that an immune co-expression network derived from cervical cancer also stratified survival probability in HNSCC [6]. To determine whether HNSCC-derived survival-associated co-expression networks could also stratify survival probability in other cancers, we tested the HNSCC WGCNA-derived module scores in cervical cancer, melanoma and lung cancer (Fig. 1A). The majority of HNSCC-derived WGCNA scores were also significant associated with survival in other cancers, although to a lesser extent (Fig. 7A, B). In both cervical cancer and lung cancer, 6 of 8 IS scores, and 7 of 8 DS scores, were significantly associated with survival. In melanoma, only 1 IS score, the immune module-derived Cyan_IS score, and 6 of 8 DS scores, were significantly associated with survival. All tested cancer types had a significant survival association with the Immune Suppression Score, and this significance was highest in melanoma (Fig. 7B).

## Discussion

The availability of large transcriptomic cancer data sets and associated clinical outcome serves as a powerful tool to identify survival-associated gene expression patterns, thereby advancing knowledge of heterogeneity in cancer biology and building frameworks for patient stratification. However, the majority of studies to date used biased approaches to interrogate survival association of specific signaling pathways and genes. To gain a better overall understanding of the interconnectedness of survival-associated genes, we used a systems biology approach based on weighted gene co-expression to decipher networks that are associated with clinical outcome. Using the TCGA HNSCC gene expression data of 519 patients, and assessing all available 20,530 genes, we identified 5129 survival-associated genes arranged into distinct gene co-expression networks. Particularly interesting, we identified 21 immune suppressive genes for which high expression was associated with increased survival. Many of the identified networks, including the immune suppressive genes, also associated with survival in cervical cancer, melanoma and lung cancer. This data represents a resource to enable the identification of novel genes and metabolic pathways that may allow patient stratification and development of new cancer-specific therapy.

HNSCC is a diverse group of cancers affecting different anatomical regions of the oral cavity and throat. HNSCC cancers can be found on the tongue, in tonsils, in the larynx or oropharynx, at the floor of the mouth and other anatomical sites. Traditionally, HNSCC is associated with a long-term habit of smoking and drinking. An increasing proportion of HNSCC, however, is associated with persistent infection with oncogenic HPVs, and it is acknowledged that HPV+ HNSCC have a better prognosis than HPV− HNSCC [30]. This was confirmed in the current study, where HPV+ HNSCC defined by either presence of HPV viral sequences or p16 expression (as a recognized surrogate for HPV-associated cancer) was correlated strongly with survival. HPV status was in fact the dominant clinicopathological trait that correlated with survival-associated gene co-expression networks. Specifically, gene networks where high expression was associated with increased survival were highly correlated with HPV+ HNSCC, and gene networks where high expression was associated with decreased survival were highly correlated with HPV− HNSCC. In contrast, tobacco, alcohol use, or clinical and pathology stage, did not correlate with the survival-associated gene co-expression networks, and future studies exploring gene-environment associations may inform these relationships more specifically [31]. Of note, this study was not powered to determine the effect of different HPV genotypes on survival-associated gene co-expression networks, as 82% of HPV+ samples expressed viral sequences of HPV16, and very few samples (*n* = 13) expressed other HPV genotypes [16].

We describe in detail three survival-associated gene co-expression networks in the current study; an immune module, an EMT module and a module rich in transcription factors and metabolic proteins. Immune-infiltrated HNSCC tumors have a better prognosis than HNSCC without immune infiltrate [32], and previous single-cell RNA sequencing studies of small cohorts have established the differences in immune lineages and cell–cell interactions between HPV− and HPV+ HNSCC [33]. Our study established 638 co-expressed immune-related genes that associate positively with prognosis. This network is enriched with genes indicative of T cell activity, natural killer-mediated cytotoxicity, and with immune suppressive genes including *FOXP3*, *CTLA4*, *IL10*, *LGAL59* (Galactin-9), *CD244* (2B4), *TIGIT*, and *TNFRSF4* (OX40-R). Furthermore, interrogating a knowledge-based list of immunosuppressive genes, we identified 21 genes, including inhibitory checkpoints such as *BTLA*, *TNFRSF17* (BAFF-R) and *TNFRSF4*, where high expression was associated with increased survival probability. Thus, paradoxically, immune-cell infiltrated HNSCC that co-expresses high levels of some immune-inhibitory gene transcripts have a better survival prognosis than HNSCC without any immune activity, regardless of whether the gene expression profile would be predicted to be immune stimulating or immune inhibitory. This observation is in line with our previous study in cervical squamous cancers, where we identified many of the same immune suppressive genes that were positively associated with survival [6]. Furthermore, we built an Immune Suppression Score based on the HNSCC-derived survival-associated immune suppressive genes, and this score also stratified survival outcome in cervical cancer, melanoma and lung cancer, where in all three cases, a high score was associated with increased survival.

The Pink EMT module consisted of 461 gene transcripts for which high expression was associated with decreased survival probability. Central pathways of this module were myogenesis, EMT and hypoxia. Our data demonstrated that HPV− tumors had a significantly higher Pink EMT score compared to HPV− tumors. This is in line with the observation that HPV− HNSCC is less radiosensitive, where hypoxia is a recognized factor that reduces radiosensitivity [34]. We acknowledge that gene expression does not always correlate with protein expression, and that the TCGA gene expression data do not offer spatial or cellular resolution for a more concise analysis of inter-cellular signaling in space. We therefore integrated HNSCC single-cell transcriptomic data to identify specific cell populations that are enriched with specific survival-associated gene co-expression networks, and identified that gene expression of the EMT module was dominantly enriched in CAFs with contractile properties (myofibroblasts), confirmed by expression of the fibroblast-activation protein (*FAP*) gene in the EMT module. This observation is consistent with an observed higher density of CAFs in HNSCC, associated with advanced tumor stage, nodal infiltration, clinical stage, vascular invasion and poor differentiation [35]. CAFs develop alongside epithelial cancer cells and acquire an activated phenotype [36], and persistently activated CAFs produce extracellular matrix components, resulting in compartmentalization of tumor nests that hinder immune cell infiltration. Indeed, we observed negative correlations between proportions of CAFs and T cells, and between a CAF score and the immune score, suggesting that one may lead to the exclusion of the other. CAFs can originate from epithelial cancer cells by transdifferentiating into myofibroblasts under oxidative stress. CAFs can also originate from endothelial cells, mesenchymal stem cells or adipocytes, and hence represent a heterogeneous group of cells that can drive EMT, invasion and metastasis [37]. CAFs also produce chemokines and cytokines, recruiting and polarizing immune cells towards immune suppression. This might explain why a high Immune Score, overall associated with increased survival, was not strongly negatively correlated with a high EMT score in our analysis. Targeting CAFs through *FAP* has shown anti-tumor efficacy in preclinical head and neck cancer models [38], and a number of FAP-targeting clinical trials in solid tumors are currently ongoing.

We further detected a large gene co-expression module containing 1565 genes (the WGCNA DarkOrange_DS genes) for which high expression was associated with decreased survival. Expression of these genes occurs in many cancer-promoting pathways including *MYC*, hypoxia, biosynthesis (ribosomal subunits) and catabolism (proteasome subunits and essential autophagy genes). Part of this module was also the central tumor-promoting cytokine gene *TGFB1*. *MYC* is one of the most commonly amplified genes in human cancers [39], and has been described as a “most wanted” target for cancer therapy. MYC proteins are implicated in a range of tumor-promoting activities including cell proliferation, dedifferentiation and stemness, angiogenesis, migration, invasion, immune evasion and therapy resistance [28]. Copy number alterations of *MYC* have also been observed in a significant proportion of HNSCC [40], but there is little research into the contribution of *MYC* genes in HNSCC. Several attempts to develop a MYC inhibitor as a broad therapeutic for the treatment of multiple cancers are ongoing, with promising preclinical results. Omomyc, a *MYC* mutant that has been delivered as transgene in cell lines, and as miniprotein in preclinical tumor models, has been shown to block proliferation and induce apoptosis of cancer cells specifically, leading to immune cell infiltration [28], and might represent an attractive target for HNSCC therapy.

The significant number of proteasome subunit genes together with *ATG5, ATG12*, *BECN1,* and VPSA genes in the WGCNA DarkOrange_DS gene list further points towards autophagy as a pathway associated with decreased HNSCC survival. Autophagy in the context of cancer is understood as double-edge sword in that it can aid cancer cell survival by removing excess potentially toxic protein aggregates stemming from enhanced metabolism, while prolonged activation of autophagy might lead to cellular self-degradation [41]. Autophagy is induced by cellular stress including hypoxia and DNA damage, and aims to control cellular damage. In a nutrient-deficient environment, however, autophagy can lead to an increase of nutrient availability by facilitating catabolism and promoting angiogenesis, thereby promoting tumor progression. Tobacco and areca nut extract contribute to HNSCC and induce oxidative stress and autophagy, and autophagy appears to play a tumor-protective role in hypoxic areas of HNSCC [42]. Autophagy is increased by hypoxia-induced expression of the DNA-damage-inducible transcript 4 (*DDIT4*) through repression of mTORC1, and consistent with this observation, *DDIT4* is part of the WGCNA DarkOrange_DS score.

To explore parallels of HNSCC-derived survival-associated co-expression networks and other cancers, we tested the HNSCC-derived WGCNA scores in cervical cancer, melanoma, and lung cancer. The majority of scores were also significantly associated with survival in cervical cancer and lung cancer, although in general, with lower significance. Melanoma produced the least range of overlap, and in particular HNSCC-derived scores associated with increased survival were not significantly survival associated in melanoma, whereas HNSCC scores associated with decreased survival were also associated with decreased survival in melanoma. We observed the closest overlap between all cancer types for the Cyan_IS Immune Score and the Immune Suppression Score, highlighting that high expression of a range of immune suppression mediators predicts better outcomes for cancer than the absence of expression of immune function genes.

In summary, this study provides a comprehensive and statistically well-powered resource of survival-associated gene co-expression networks in HNSCC, its interrelations and relations with HPV status, offering new insights into the heterogeneity of HNSCC disease and parallels with other cancer types. We acknowledge that gene expression does not always correlate with protein expression, and that the TCGA gene expression data do not offer spatial or cellular resolution for a more concise analysis of inter-cellular signaling in space. However, this study describes patterns of survival-associated networks, which provide a powerful tool to start understanding the interconnectedness of signaling pathways within the tumor. It is important to recognize that the delicately regulated tumor ecosystem and individual heterogeneity cannot be oversimplified. Hence, once reaching statistical power, new technologies with single-cell and spatial resolution will in time improve delineation of signaling systems that determine outcome.

## Supplementary information

Supplementary File 1

## Data Availability

All data are available in this manuscript and its associated supplementary files. Custom computer code can be provided upon request to the corresponding author.
